# Blue carbon gains from glacial retreat along Antarctic fjords: What should we expect?

**DOI:** 10.1111/gcb.15055

**Published:** 2020-03-23

**Authors:** David K. A. Barnes, Chester J. Sands, Alison Cook, Floyd Howard, Alejandro Roman Gonzalez, Carlos Muñoz–Ramirez, Kate Retallick, James Scourse, Katrien Van Landeghem, Nadescha Zwerschke

**Affiliations:** ^1^ British Antarctic Survey NERC Cambridge UK; ^2^ University of Ottawa Ottawa ON Canada; ^3^ Falmouth Campus University of Exeter Penryn UK; ^4^ Facultad de Ciencias Centro de Investigación en Biodiversidad y Ambientes Sustentables (CIBAS) Universidad Católica de la Santísima Concepción Concepción Chile; ^5^ Instituto de Entomología Universidad Metropolitana de Ciencias de la Educación Santiago Chile; ^6^ Ocean Sciences Bangor University Bangor UK

**Keywords:** Blue carbon, climate change, fjord, glacier retreat, sequestration, Southern Ocean

## Abstract

Rising atmospheric CO_2_ is intensifying climate change but it is also driving global and particularly polar greening. However, most blue carbon sinks (that held by marine organisms) are shrinking, which is important as these are hotspots of genuine carbon sequestration. Polar blue carbon increases with losses of marine ice over high latitude continental shelf areas. Marine ice (sea ice, ice shelf and glacier retreat) losses generate a valuable negative feedback on climate change. Blue carbon change with sea ice and ice shelf losses has been estimated, but not how blue carbon responds to glacier retreat along fjords. We derive a testable estimate of glacier retreat driven blue carbon gains by investigating three fjords in the West Antarctic Peninsula (WAP). We started by multiplying ~40 year mean glacier retreat rates by the number of retreating WAP fjords and their time of exposure. We multiplied this area by regional zoobenthic carbon means from existing datasets to suggest that WAP fjords generate 3,130 tonnes of new zoobenthic carbon per year (t zC/year) and sequester >780 t zC/year. We tested this by capture and analysis of 204 high resolution seabed images along emerging WAP fjords. Biota within these images were identified to density per 13 functional groups. Mean stored carbon per individual was assigned from literature values to give a stored zoobenthic Carbon per area, which was multiplied up by area of fjord exposed over time, which increased the estimate to 4,536 t zC/year. The purpose of this study was to establish a testable estimate of blue carbon change caused by glacier retreat along Antarctic fjords and thus to establish its relative importance compared to polar and other carbon sinks.

## Graphical abstract

As glaciers retreat, they open up new space in fjords for life to store carbon, generating a negative feedback on climate change. This is unquantified, so we tried to derive a testable estimate of glacier retreat‐driven blue carbon gains by investigating three West Antarctic Peninsula (WAP) fjords. We multiplied mean glacier retreat rate by regional zoobenthic carbon means to suggest generation of 3,130 tonnes of new zoobenthic carbon per year (t zC/year). We tested this by capture and analysis of 204 seabed images along WAP fjords, which increased the estimate to 4,536 t zC/year.

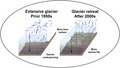

## 1 INTRODUCTION

Declarations of ‘climate emergency’ and more urgent aim at developing carbon neutral economies have drastically increased interest in carbon capture, storage and sequestration. Saban, Chapman, and Taylor (2018) have shown that global greening, and thus potential carbon capture, have increased with rising atmospheric CO_2_ levels but what of storage and sequestration? Blue carbon (held within marine organisms) is prolific in capture and efficient in sequestration rate to the extent that Duarte, Middelburg, and Caraco, (2005) estimate blue carbon to be responsible for 50% of all oceanic carbon burial. Typical blue carbon habitats, such as mangrove swamps, seagrass beds and salt marshes are declining across global habitats, for example the International Union for the Conservation of Nature estimates ~7% per year for seagrasses (see https://www.iucn.org/content/seagrass-habitat-declining-globally), making them essentially positive feedbacks on climate change. Very little is known about blue carbon in the polar regions where mangroves, salt marshes and seagrasses are absent. Fjords with marine terminating glaciers can be highly productive (e.g. in the Arctic, Meire et al., 2017) and accumulate considerable fjord floor carbon (e.g. in the Antarctic, Grange & Smith, 2013). It is becoming clearer that climate‐mediated losses of marine ice over high latitude continental shelf areas are a rare, valuable negative feedback on climate change, albeit globally small in magnitude (Barnes, 2017; Barnes, Fleming, Sands, Quartino, & Deregibus, 2018). As many glaciers are retreating from Antarctica's fjords, the newly emerging seabed creates a brand new habitat for primary and secondary production and this acts to counter the present effects of climate change. Blue carbon (produced by marine biological activity) standing stock and production are high in shallower water but require the sediments, usually associated with low energy habitats, for burial and ultimate sequestration.

Antarctica is the only continent with no open–water, nearshore low energy environments. The West Antarctic Peninsula (WAP) coast, however, has many ice‐filled small fjords, which are progressively opening up due to glacier retreat. They are probably playing an important and increasing role in carbon sequestration which is little evaluated. There are ~240 glaciers along the WAP of which nearly 90% (=216) are now retreating, and their retreat rates are increasing (Cook et al., 2016). Given the importance of natural carbon sinks which involve genuine sequestration and the rarity of negative feedbacks it would appear crucial to evaluate an emerging one in Antarctica's opening fjords (Grange & Smith, 2013). Is lack of quantification of fjords' role as an increasing capacity for carbon sink important as a source of uncertainty for climate models? Marine ice losses comprise multiple sources; sea ice (such as fast ice), ice shelves and glaciers. For seasonal sea ice losses and ice shelf disintegration, the quantification of blue (marine biological) carbon has been attempted (Barnes, 2017; Barnes et al., 2018) and is ongoing with the Changing Arctic Ocean Seafloor project throughout the Barents Sea. For glacial retreat, a third source of marine ice loss, the calculations for carbon sink capacity are problematic, as previous work has estimated average retreat rates rather than areas of glacier lost (=habitat gained; Cook et al., 2016). Here we erect a testable estimate of WAP fjordic blue carbon gains by calculating areas of glacier lost and fitting existing regional blue carbon data to it. The regional blue carbon data we used to do this was zoobenthic seabed carbon values from the geographically closest analogous environments; fjords retreating at South Georgia (in Barnes, 2017).

## 2 METHODS; HOW TO ESTIMATE EMERGING FJORDIC BLUE CARBON?

**FIGURE 1:**
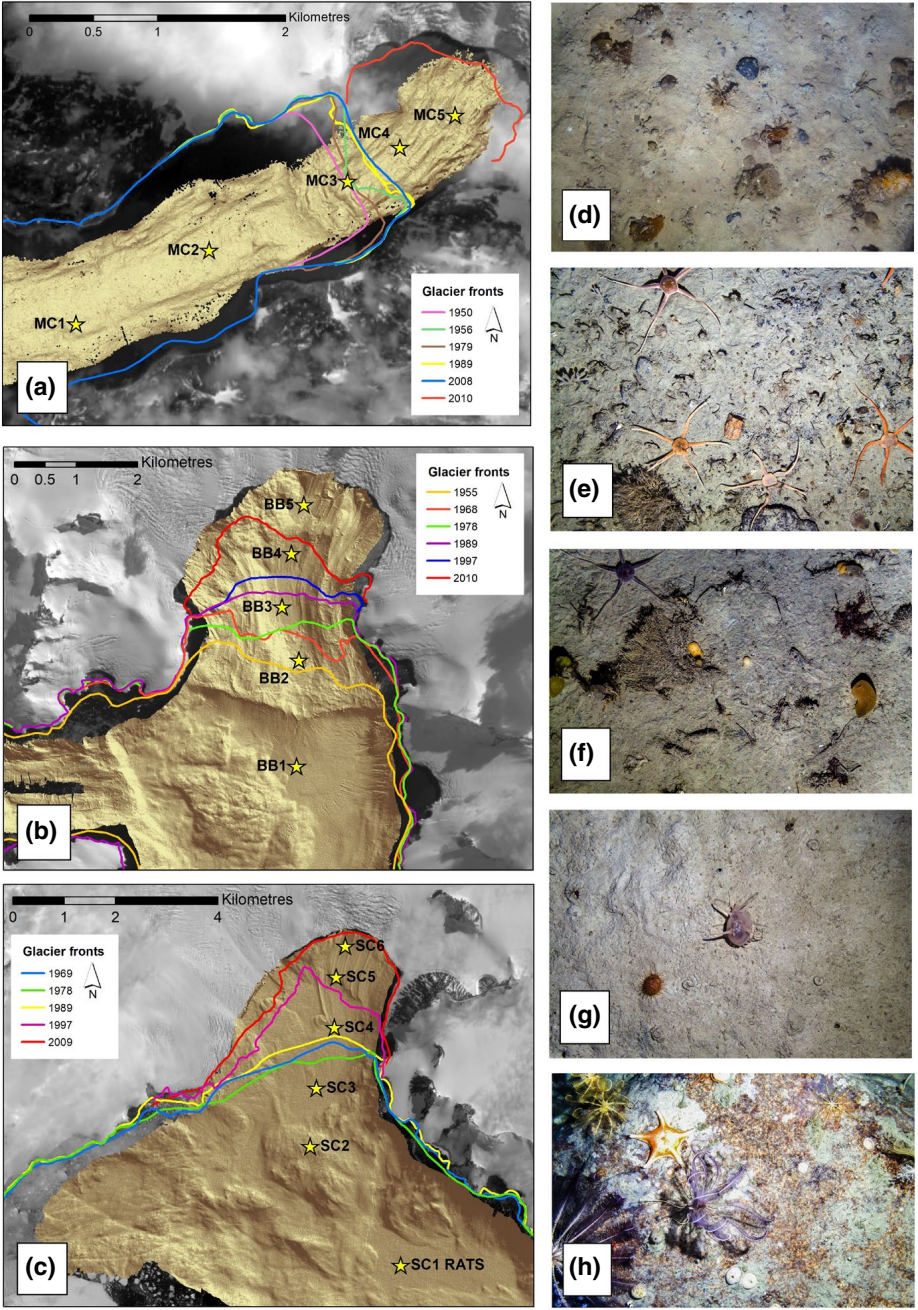
Glacier retreat lines and examples of blue carbon in seabed assemblages along the West Antarctic Peninsula. Position of shelf underwater camera system sampling stations, glacier retreat positions and seabed biota of three fjords along the West Antarctic Peninsula. The fjords are Marian Cove (a), Börgen Bay (b) and Sheldon Cove (c). Seabed biota from vertical camera images at 68–127 m depth at inner fjord (d), moraine (e) outer fjord (f) as well as typical shelf (non fjord, g) and rich drop stone habitats (h)

The work presented here attempted to derive a testable estimate for seabed biological carbon gains as a result of recent rapid glacier retreat along selected WAP fjords (see map in Figure S1). Firstly we calculated the area of fjord emergence (=glacier loss) from literature data (Cook et al., 2016; georeferenced shape files in ArcGIS) of glacier fronts for three study fjords (Figure 1). The study fjords were Marian Cove (King George Island), Börgen Bay (Anvers Island) and Sheldon Cove (Adelaide Island). These have retreated 1.71, 7.8 and 7.8 km^2^ from 1978/79 to 2019, and as such are representative of WAP glaciers (Cook et al., 2016). Mean annual glacier area loss rates for Marian Cove, Börgen Bay and Sheldon Cove since 1978/79 were 0.042, 0.191 and 0.191 km^2^/year. We then mapped the seabed of each fjord using multibeam swath (using Kongsberg EM122) and collected images of the seabed at multiple distances (sites in Figure 1; Figure S2). We multiplied recently emerged area by blue carbon literature data per unit area for each of the three fjords to generate estimated *X* tonnes carbon km^2^/year. New areas of fjord are recorded as starting to emerge in 1950–1970 (Cook et al., 2016), but change has been non–linear and varies between fjords (glacier front by year is shown in Figure 1a–c). The literature data we used were Inner fjord environments at South Georgia, which typically generate 0.4 (muds), 3.7 (moraines) and 17.4 (shallows and walls) tonnes immobilized carbon, km^2^/year (Barnes, 2017). This assumes that newly emerging fjords along the WAP would have similar blue carbon content to retreating glaciers around South Georgia, but there is currently little literature on succession in benthic carbon standing stock with glacier retreat time. Assessment of megafaunal patterns along two WAP fjords (Grange & Smith, 2013; Sahade et al., 2015) suggests intra‐region variance may be as considerable as that between regions, at least with respect to composition of biota. The mean carbon value that we derived across our three study areas was then multiplied by the number of retreating fjords along the WAP (216; see Cook et al., 2016).

We tested this initial estimate by capturing and analysing 204 high‐resolution seabed images (each 405.7 × 340.6 mm, 12 MB, 5 MegaPixel) along study fjords from research cruise JR17001 (2017). Images were analysed for the density of each of the 13 functional groups of benthos (as per Barnes, 2017). The 13 function groups were defined as follows: suspension feeder pioneers (A), climax suspension feeders (B), sedentary suspension feeders (C), mobile suspension feeders (D), deposit feeding crawlers (E), deposit feeding vermiform (F), deposit feeding, shelled burrowers (G), calcareous grazers (H), scavenger/predator, sessile soft bodied (J), scavenger/predator, sessile calcareous (K), scavenger/predator, mobile soft bodied (L), scavenger/predator, mobile calcareous (M), scavenger/predator, arthropod (N) and flexible strategy (P). We fitted the resulting density of functional group data to the following model:

The model used to give carbon in g/m^2^ (=tonnes/km^2^) was, per shelf underwater camera system image = ((0.06*A) + (0.11*B) + (0.39*C) + (0.18*D) + (0.13*E) + (0.15*F) + (0.5*G) + (0.17*H) + (0.11*J) + (0.12*K) + (0.25*L) + (0.45*M) + (0.13*N) + (0.16*P)).

This model showed a good fit to existing zoobenthic blue carbon data (Barnes, 2017) at South Georgia and South Orkney (*r* ^2^ = 64%, *F* = 682, *p* < .001).

Glaciers contain approximately 0.02–0.04 mg carbon/L (Legrand et al., 2013), which we converted to 18.3–36.7 t km^3^. This is exported from fjords with ice calving and the fate of this carbon is uncertain, so we have conservatively assumed this as recycled with zero sequestration. Thus to estimate carbon losses per fjord we multiplied the approximate volume of ice lost from ~1980 to 2017 by carbon content of ice per fjord. The mean value of this was scaled up by the number of retreating fjords (216), which in turn was multiplied by 10 to give value per decade. Finally we subtracted the maximum carbon within glacier ice loss from minimum estimated blue carbon gains and minimum carbon within glacier ice loss from maximum estimated blue carbon gains to derive estimated net carbon change along WAP fjords.

## 3 RESULTS; HOW MUCH NEW SEABED ZOOBENTHIC CARBON OCCURS IN EMERGING ANTARCTIC FJORDS

Areas of glacial retreat and seabed exposure are shown for three study fjords in Table 1 (and Figure 1).

**TABLE 1:** New habitat exposed from glacier retreat along three Antarctic fjords. Data sources are glacier retreat positions with time (shown in Figure 1, from Cook et al., 2016), seabed topography from multibeam data (see Figure S2, data available from UK Polar Data Centre)

Fjord	Glacier retreat area 1978/79−2019/km^2^	Fjord floor mud exposed	Fjord floor moraine exposed	Fjord sides exposed
Marian Cove	1.71	1.65	0.08	0.4
Börgen Bay	7.81	7.5	0.4	1.0
Sheldon Cove	7.82	7.9	0	0.6

We estimated 8–22 t zC/year has been generated at the three study fjords (mean per fjord 14.5 t zC/year; Table 2). Multiplied by all WAP fjords (216), this totalled 3,130 t zC/year. This ~3,000 t zC/year thus scales to ~31,300 tonnes of zoobenthic carbon per decade (t zC/decade).

**TABLE 2:** Blue carbon in habitat exposed from glacier retreat along three Antarctic fjords. Literature amounts of blue carbon per habitat are from Barnes (2017), and the areas are from Table 1

Fjord	Fjord floor mud carbon	Fjord floor moraine carbon	Fjord sides carbon	Fjord floor carbon totals (t/year)
Literature blue carbon data	0.4 t km^2^/year	3.7 t km^2^/year	17.4 t km^2^/year	
Marian Cove	1.71 × 0.4	0.1 × 3.7	0.4 × 17.4	8.0
Börgen Bay	7.5 × 0.4	0.4 × 3.7	1 × 17.4	21.9
Sheldon Cove	7.9 × 0.4	0 × 3.7	0.6 × 17.4	13.6
Mean for three study fjords				14.5
Total for 216 fjords				3,130

Analysis of the high‐resolution seabed images showed that close to the glacier terminus (exposed over the last decade) the epibiota seen on images were typically high sedimentation tolerant pioneers, such as *Cnemidocarpa verrucosa* (Figure 1d). Older, outer sediment basins (exposed for a few decades) had a denser more varied fauna (Figure 1f). Exposed hard substrata, such as glacial moraines were richer, with higher biomass, and likely to sequester this due to nearby surrounding sediment basins (Figure 1e). Offshore WAP shelf (G) where there are occasional ice rafted dropstones (H) are shown for context. We multiplied the mean density of each functional group of zoobenthos to mean carbon per group using a model derived from regionally ground truthed data (Barnes, 2017). We found that study–fjord seabeds which had newly emerged from retreating glaciers may gain about 12–31 t zC/year. Multiplying fjord mean image derived values to total WAP glacier numbers generated an estimate of WAP fjordic zoobenthic carbon storage at 4,536 t zC/year (Table 3). Per decade this would approximate to 45,360 t zC. About a quarter of this would be expected to be sequestered, which would be 7,375 and 11,627 tonnes (for the theoretical [Table 2] and seabed image‐based [Table 3] estimates respectively).

**TABLE 3:** Blue carbon in habitat exposed from glacier retreat along three Antarctic fjords from functional group densities per seabed image

Fjord	Fjord floor mud carbon	Fjord floor moraine carbon	Fjord sides carbon	Fjord floor carbon totals (t/year)
Marian Cove	1.71 × 2.7	0.1 × 4.5	0.4 × 17.4	12.03
Börgen Bay	7.5 × 1.7	0.4 × 2.3	1 × 17.4	31.07
Sheldon Cove^a^	13.6 × 1.46			19.9
Mean for three study fjords				21.0
Total for 216 fjords				4,536

^a^ No shelf underwater camera system images were captured in Sheldon Cove. Data were generated by multiplying the original estimate by average increase (×1.46) as other two fjords.

The ice volume calved and thus carbon potentially exported, from carbon held within glaciers are shown in Table 4. Per decade this would be 1476–2938 t zC, which is about 0.3%–1% of the theoretical and imaged based carbon estimates of blue carbon gains from glacier retreat. Subtracting such losses from estimated gains (Tables 2 and 3) gave net balances of 28,362–43,844 t zC gain per decade.

**TABLE 4:** Carbon encased in glacier ice, exported from glacier retreat along three Antarctic fjords from literature data. Mean glacier thickness value (0.25 km) was taken from Paul (2017)

Fjord	Glacier volume (area × thickness)	Ice vol calved × carbon content/no. years	Min carbon mass lost/year (t)	Max carbon mass lost/year (t)
Marian Cove	1.71 × 0.25 = 0.38	(0.43 × 18.33 to 36.67)/38	0.21	0.41
Börgen Bay	7.5 × 0.25 = 2.39	(1.88 × 18.33 to 36.67)/38	0.9	1.8
Sheldon Cove	7.8 × 0.25 = 1.8	(1.95 × 18.33 to 36.67)/38	0.94	1.88
Mean for three study fjords			0.68	1.36
Total for 216 fjords			147.6	293.8

## 4 DISCUSSION: ANTARCTIC FJORD CARBON IMPORTANCE, ERROR LEVELS AND FUTURE TESTS

The high energy coastline around Antarctica is very different from elsewhere in the world, it is much less studied and has none of the most efficient blue carbon habitats of mangroves, saltmarshes and sea grass meadows. Amongst the most productive of these, mangrove swamps, are thought to be responsible for 10% of global carbon burial (174 g zC m^−2^ year^−1^), despite only occupying 138,000 km^2^, just 0.027% of Earth's surface (see Duarte et al., 2005). Our estimates of Antarctic fjord blue carbon efficiency are two orders of magnitude lower than reported for mangroves (mean 13.7–19.5 t zC per 6 km^2^ = 2.3–3.3 g zC m^−2^ year^−1^). This is even less than the average of ~5 g zC m^−2^ year^−1^ for shallow Antarctic shelves (Arntz, Brey, & Gallardo, 1994) but such habitats are young, still being colonized and stressed by sedimentation (Sahade et al., 2015). We scaled up our three study glaciers to the 216 retreating in the WAP region (Cook et al., 2016), but there are 14,725 marine glaciers in the wider southern polar region (Paul, 2017). Thus a considerably higher scaling factor (up to 68×) is likely to become appropriate to understanding potential blue carbon change with glacier retreat. In total the global area occupied by southern polar marine glaciers is 137,866 km^2^, a very similar proportion of Earth's surface to that occupied by mangroves. However calculations of carbon change with ice losses need to factor in release of ice‐bound carbon.

Ice holds small quantities of ice‐bound carbon, but because ice cap volume is so considerable these add up to Pg of carbon in global ice. Thus rapid recent glacier retreat has driven concern about potential carbon ‘losses’ from ice–bound carbon released into the ocean carbon cycle during glacier retreat (Legrand et al., 2013). However we calculate that for marine glaciers such ice‐bound carbon losses (Table 4) are very small (<1%) compared to blue carbon gains (Tables 2 and 3). Estimated net carbon for WAP glacier retreat generated a modest 2836–4384 t carbon gain per year equivalent to net production by ~140 ha of tropical forest. Sequestration potential of the blue carbon present is high in Antarctic fjords (Grange & Smith, 2013), compared with open shelf and especially non‐aquatic environments (Barnes et al., 2018). Thus, unlike forests, up to a quarter of zoobenthic blue carbon generated at deep continental shelf depths can be genuinely sequestered (see Barnes, Sands, Richardson, & Smith, 2019). This would mean >1,000 t zC/year just in WAP fjords. Estimated gains of blue carbon by WAP glacier retreat (<5 × 10^3^ t/year) are small compared with estimates of blue carbon gains from ice shelf losses through opening up of productive new habitat and leaving nutrient‐fertilized wakes of enhanced productivity (Duprat, Bigg, & Wilton, 2016). Giant icebergs (e.g. A68 recently calved from Larsen C) formed by shelf disintegration may generate 10^6^ t blue carbon/year (Barnes et al., 2018). Even if multiplied for all southern polar glaciers (3 × 10^5^ t carbon/year), glacier retreat would be at least an order of magnitude smaller than seasonal sea ice reductions 6 × 10^7^ t carbon/year and ice shelf losses 2 × 10^7^ t carbon/year (Barnes et al., 2018).

To consider or compare only habitat and blue carbon sink source sizes may be missing the importance of polar continental shelves. Compared with lower latitude sinks, glacier retreat and even sea ice and ice shelf losses are clearly small in carbon store and efficiency. However, unlike elsewhere blue carbon around Antarctica is increasing with climate change, and the productivity within emerging fjords is likely to further increase with age and seasonal sea ice loss (Barnes, 2017). Slight increases in sea temperature may also increase polar blue carbon but increases of 2°C or more could have varying influences (Ashton, Morley, Barnes, Clark, & Peck, 2017). Thus sea ice, ice shelf and glacier loss are, crucially, all negative feedbacks on (mitigate) climate change.

### 4.1 Error and meaningfulness of blue carbon sink comparisons

The error involved in our estimates is likely to be considerable for multiple reasons: (a) glacier retreat rates and areas differ considerably between fjords (Cook et al., 2016) making scaling difficult. (b) We only investigated three fjords, which together account for less than 2% of retreat even within the WAP. (c) Our estimates suggested blue carbon performance could differ by more than a factor of 3 across fjords. (d) Our image based estimate (Table 3) was nearly double our theoretical estimate (Table 2). (e) Our blue model was based on data from South Georgia and South Orkney Islands (Barnes, 2017) rather than from the WAP region. Similar WAP environments can be more productive (Grange & Smith, 2013; Sahade et al., 2015) by a factor of ~1.44 (Barnes, 2017). There are many assumptions implicit in our calculations, such as using mean glacier thickness (Paul, 2017). Measurements of all such factors have to be realistic for a remote, and difficult and expensive to access region.

## 5 CONCLUSIONS

Fitting existing regional blue carbon data to averaged fjord seabed area emergence rates from glacier retreat suggests that WAP fjords may generate >3,000 t zC/year. Our initial test of this theoretical estimate using seabed images from study fjords suggests that zoobenthic carbon is at a comparable, but slightly higher value of 4,536 t zC/year. Our imagery showed that most seabed biota were young pioneers so we expect the seabed carbon in these young fjords to considerably increase with fjord age as ecological succession leads to biological complexity. New fjord work elsewhere along retreat paths (e.g. at Fjord Eco: Grange & Smith, 2013; and Potter Cove: Sahade et al., 2015) should provide context of how representative our study fjords are of WAP or Antarctic fjords in general. If correct our estimated values are small in comparison with Antarctic blue carbon gains from other marine ice losses, and globally very small. However they are likely to significantly increase and have high conversion to genuine sequestration levels. Antarctic fjords emergence should increase until WAP glaciers retreat past grounding lines, but their value as carbon sinks could rise long after this until development of mature, climax benthic communities. The value of our estimates would make fjords the smallest component of marine ice loss‐related carbon sinks by an order of magnitude (compared with seasonal sea ice and ice shelves). However, the nature of fjords (steep productive sides and muddy sea floors) means their sequestration potential is likely to be high compared to the more extensive, typical continental shelf areas (Barnes, 2017). As the least known, but increasing part of one of our planet's most significant negative feedbacks on (mitigating) climate change, we argue the potential of these carbon sinks are most important to fully quantify. Testing the magnitude of polar fjordic role in carbon storage and sequestration will aid the understanding of carbon sink balances and climate change–feedback variability and could reduce uncertainty in model projections.

## Supporting information

Fig S1‐S2
