# Supplementary material for: Blue carbon gains from glacial retreat along Antarctic fjords: What should we expect?
Source: Glob Chang Biol. 2020 Mar 23;26(5):2750–5. doi: 10.1111/gcb.15055 (PMC7216916; doi:10.1111/gcb.15055)

**Supplemental information**

**Blue carbon gains from glacial retreat along Antarctic fjords: what should we expect?**

D.K.A. Barnes^1^, C.J. Sands^1^, A. Cook^2^, F. Howard^1^, A. Roman Gonzalez^3^, C. Muñoz-Ramirez^4^, K. Retallick^5^, J. Scourse^3^, K Van Landeghem^5^ and N. Zwerschke^1^

The positions of the three fjords we studied in detail (UK-Chile ICEBERGS project) are shown in figure S1. The bathymetry (from multibeam collected on ICEBERGS research cruises) is shown in figure S2.

**Supplemental figure**

Supplemental regional map of West Antarctic Peninsula study region and three fjords investigated in detail.

Fig. S1

Fig S2 The bottom topography and surrounding geography of the three study fjords, Marian Cove (top), Borgen Bay (middle) and Sheldon cove (bottom).


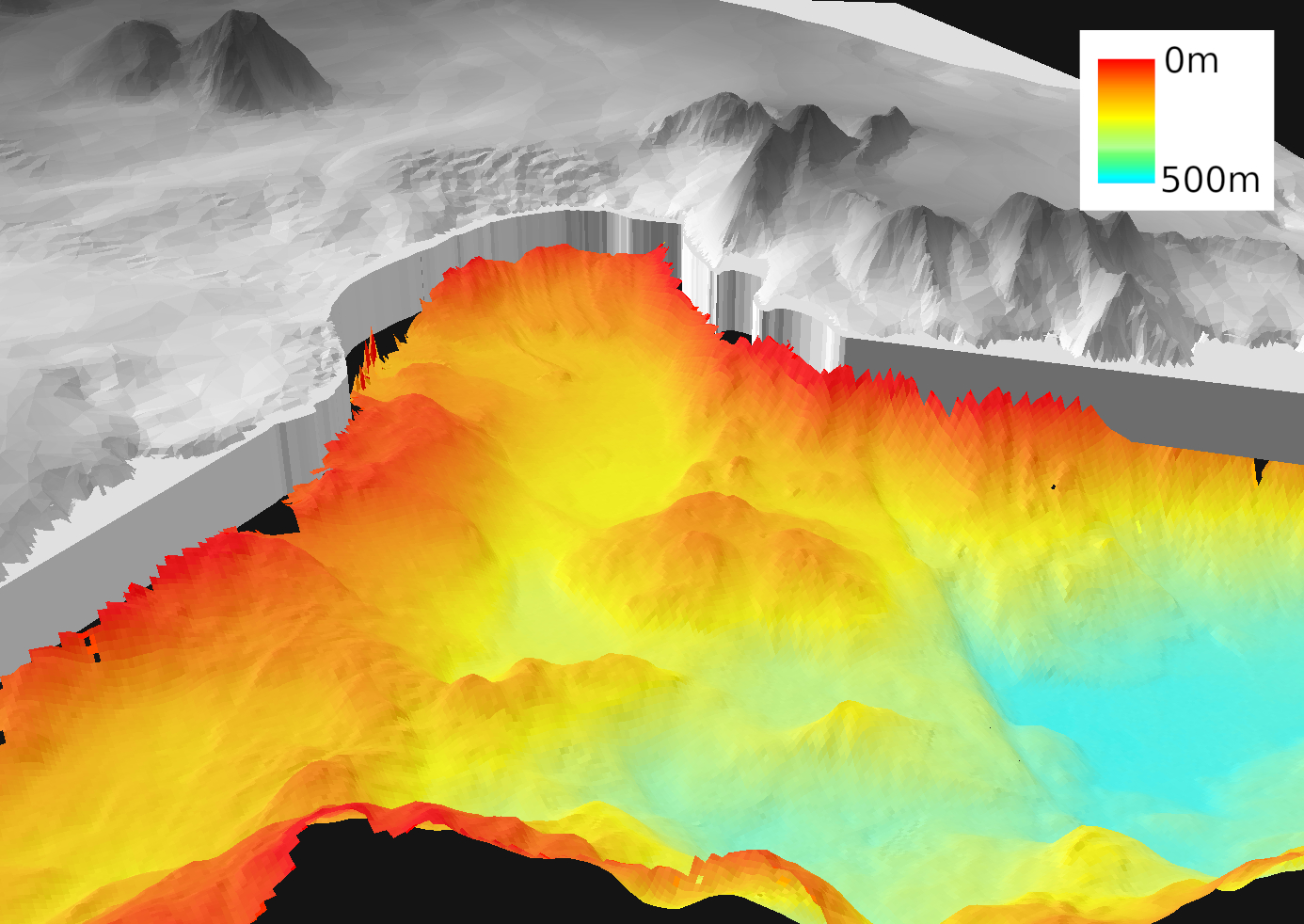

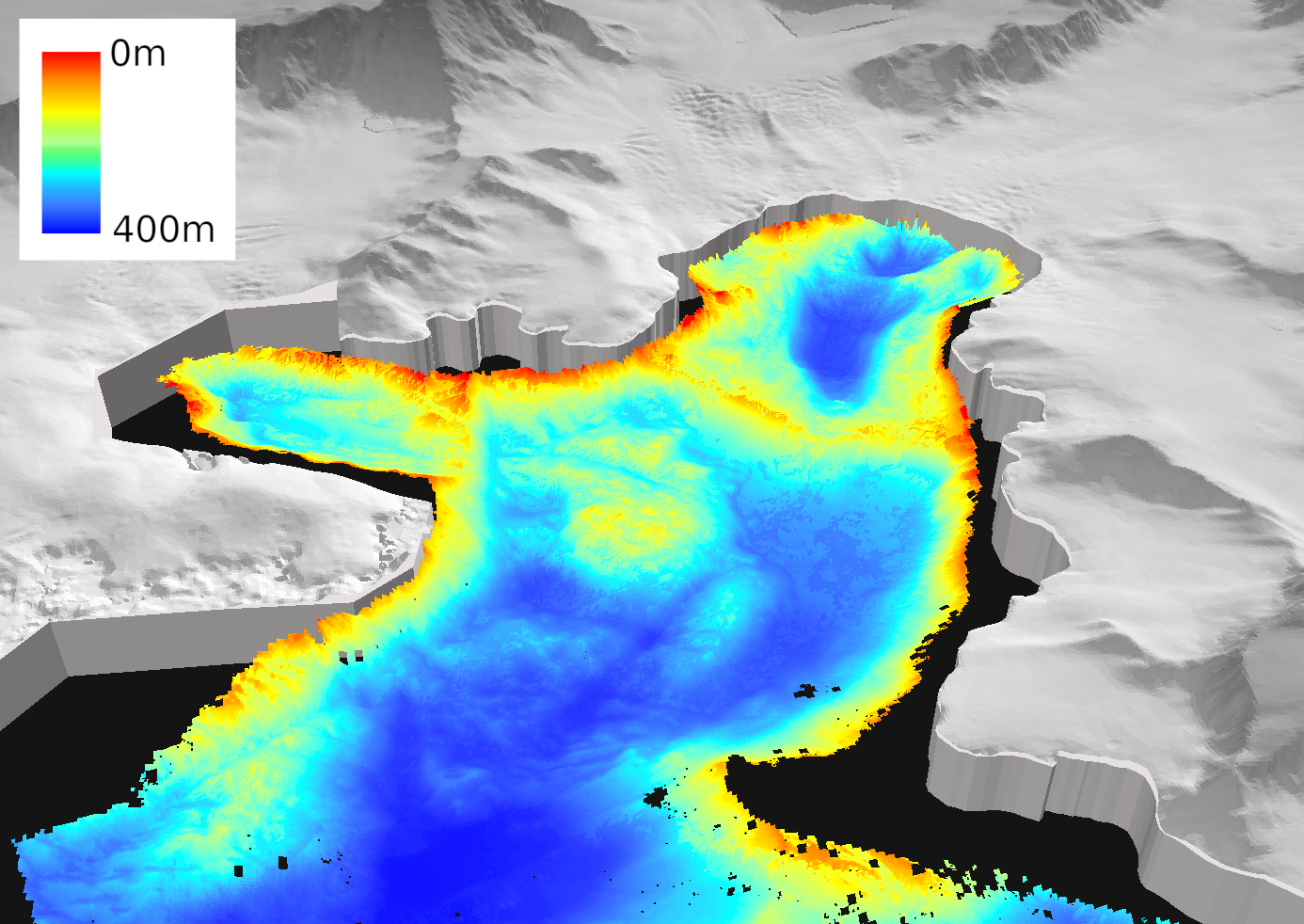

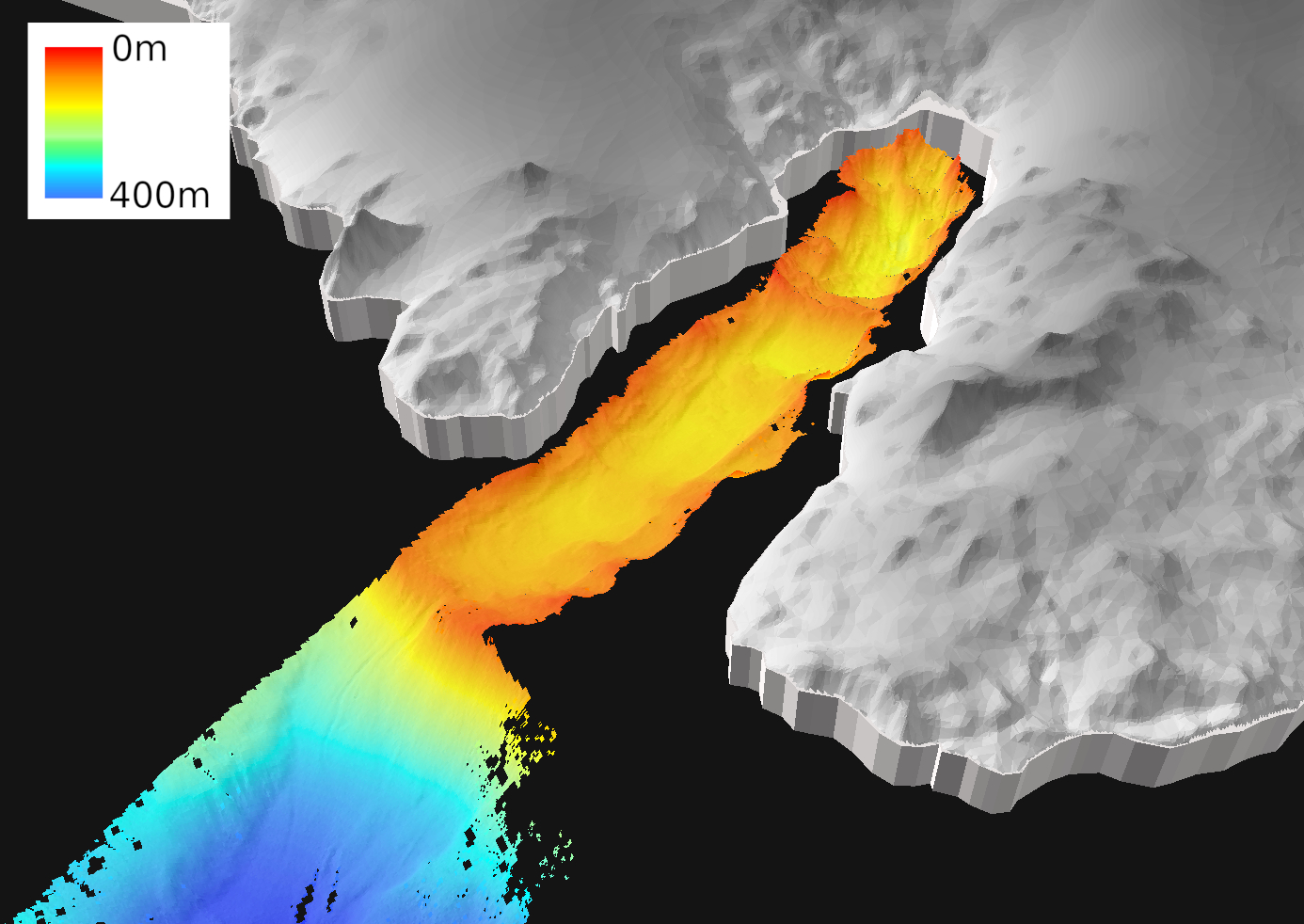

Supplement: Supplementary file 1 — Fig S1‐S2 [file GCB-26-2750-s001.docx]
